# Supplementary material for: Adaptive artificial evolution of droplet protocells in a 3D-printed fluidic chemorobotic platform with configurable environments
Source: Nat Commun. 2017 Oct 26;8:1144. doi: 10.1038/s41467-017-01161-8 (PMC5658334; doi:10.1038/s41467-017-01161-8)
Supplement: Supplementary file 1 — Supplementary Information [file 41467_2017_1161_MOESM1_ESM.pdf]

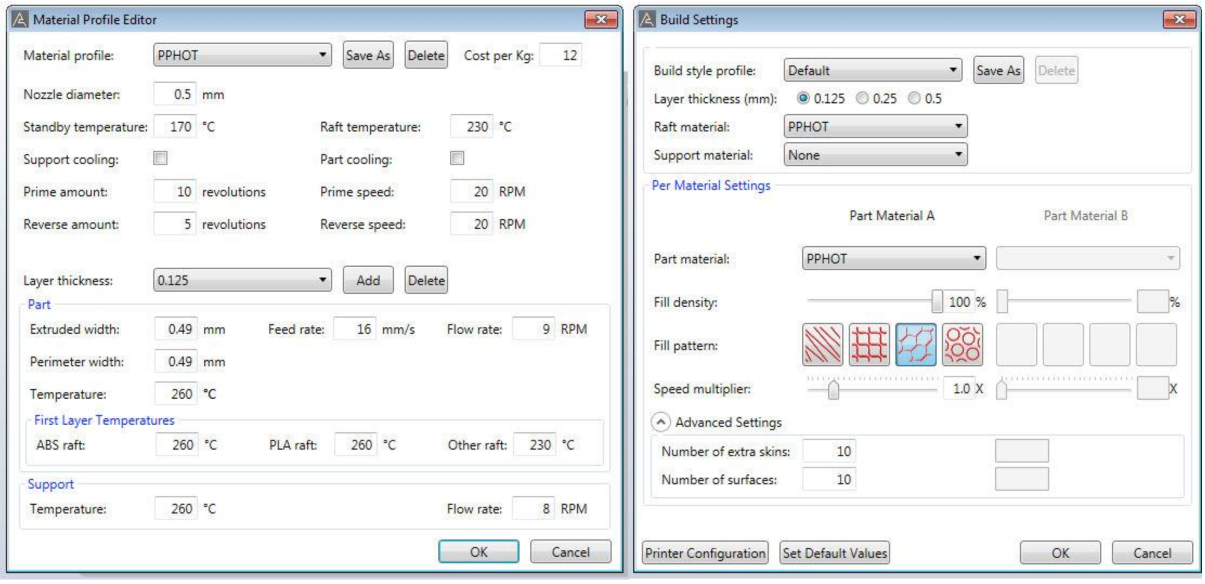

Supplementary Figure 1. Axon 2 configuration. These two screenshots detail the configuration used when manufacturing our devices using the software Axon 2 from “Bit from bytes”. The only configuration parameter we changed depending on the device was the “Flow rate”, which fluctuated between 8.5 and 10 RPM.

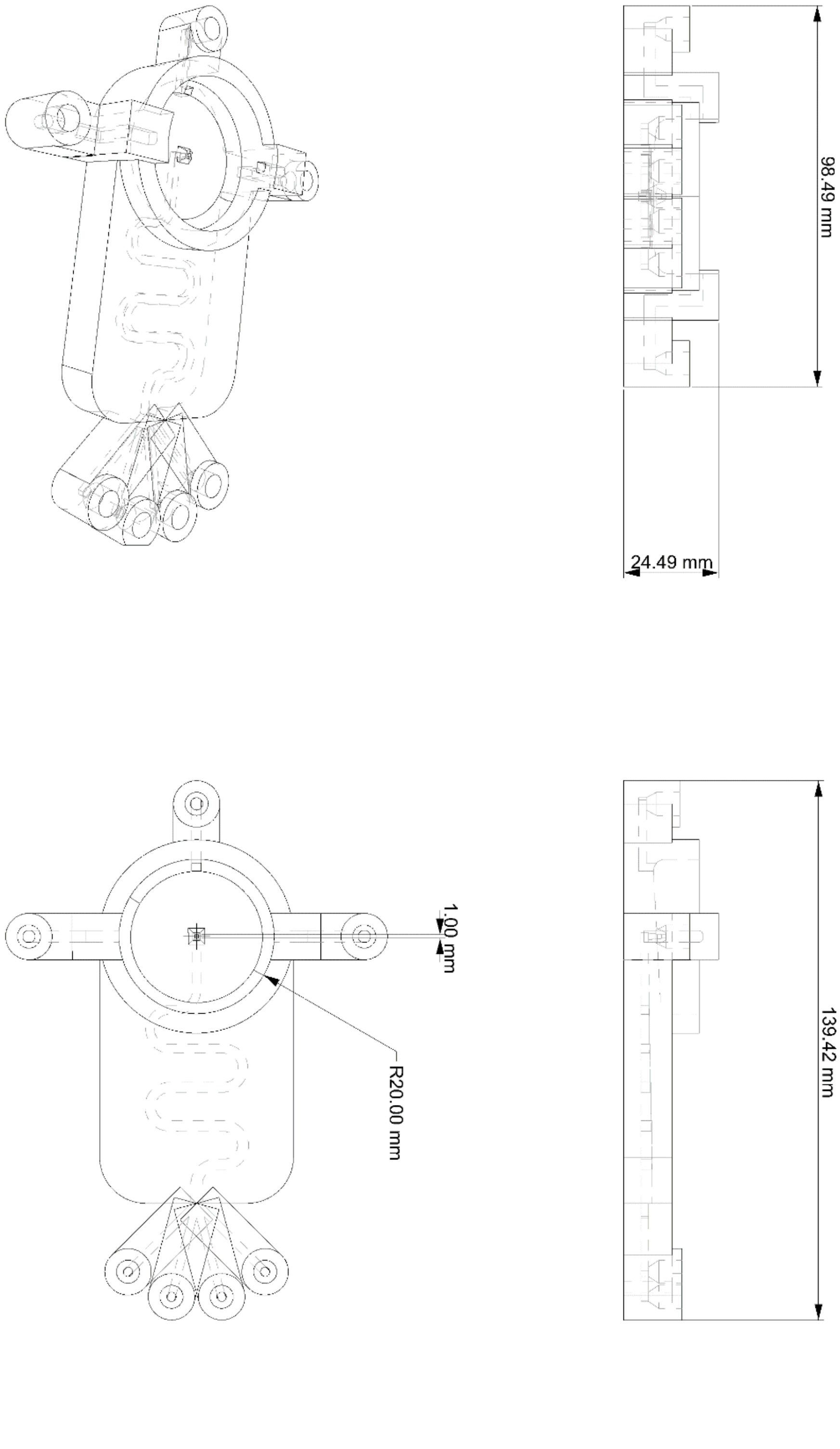

Supplementary Figure 2. CAD design of the 3D printed fluidic device. Engineering drawing of the 3D printed fluidic device generated using "Rhinoceros". Only outlines detailed. The holes used as inlets had a diameter of 6 mm in order to fit the ferrules used. The hole to generated droplets was a square with a side length of 1 mm, as detailed.

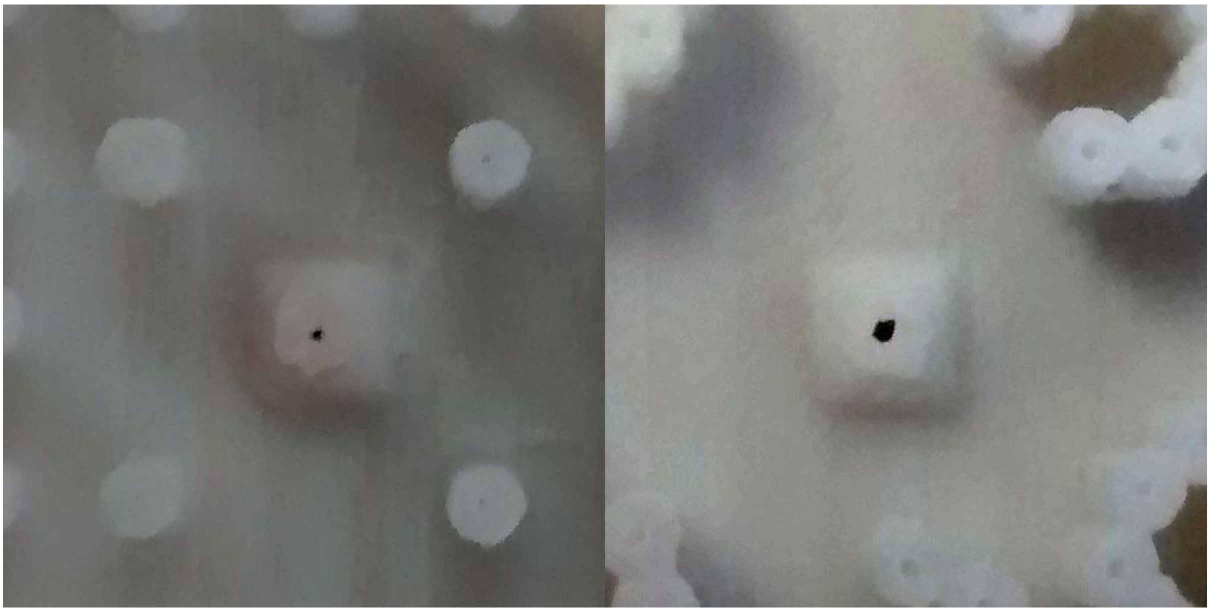

Supplementary Figure 3. Device outlet holes differences. Although both devices were printed under the same conditions, differences can be seen in the size of the outlet hole.

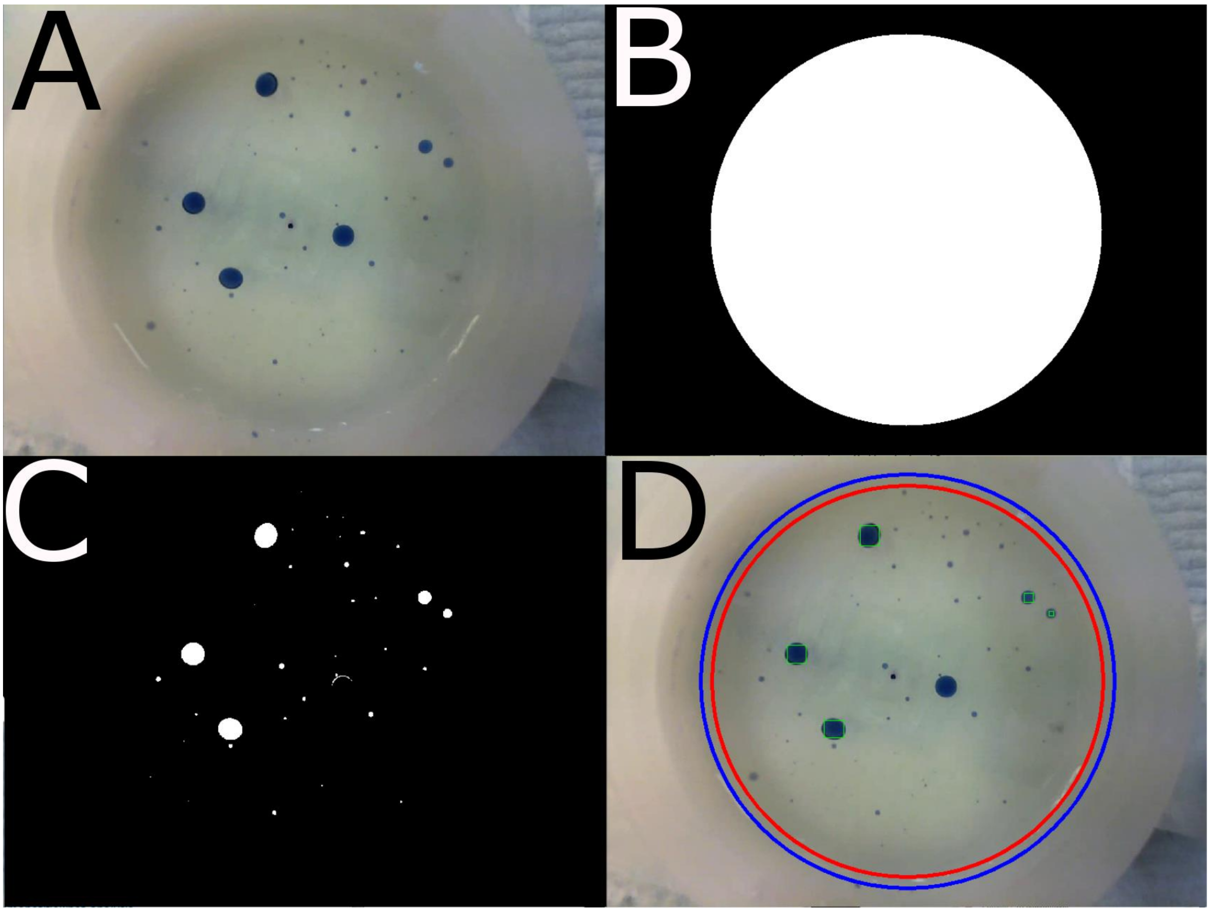

Supplementary Figure 4. Image analysis. A: Frame as received from the camera. **B:** The mask used that defined the arena. All the black pixels were ignored. This mask was always the same through all the experiments. **C:** Results from the Mixture of Gaussians background subtraction. **D:** Final result with the detected droplets. All the droplets from ``Bottom left'' which were detected there but that don't appear now were disregarded because they were not big enough, or because they were not moving.

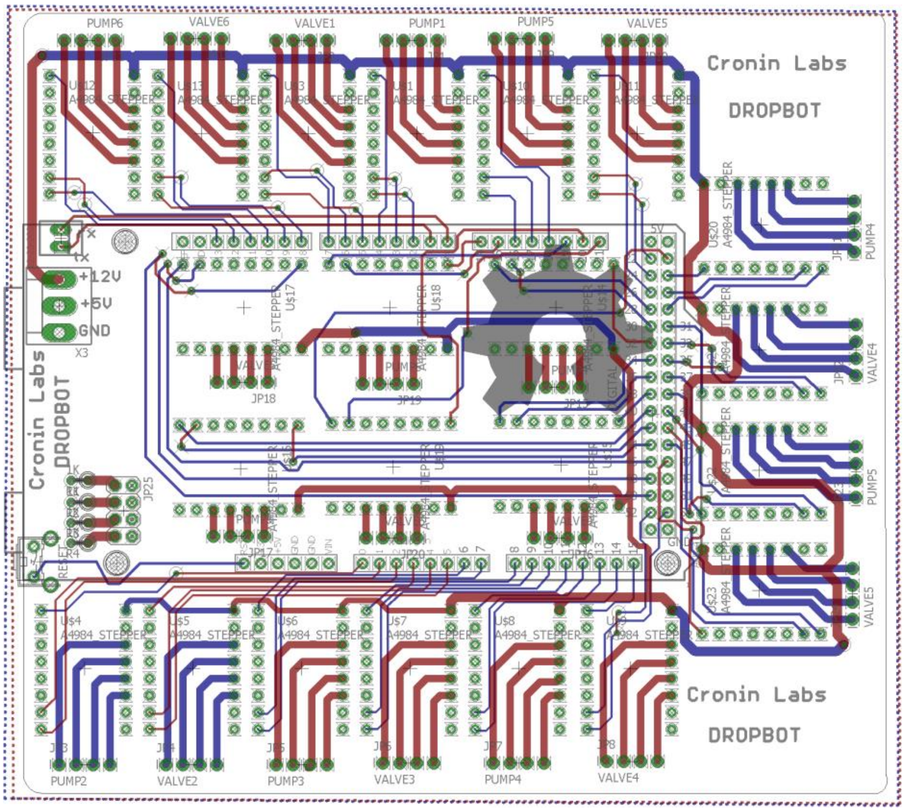

Supplementary Figure 5. Arduino Due Shield. A shield for the microcontroller board based on "Arduino Due" was developed as part of this project with the objective of maximizing the number of "Pololu A4988" drivers used. Up to 22 drivers could be used, which powered up to 11 pumps (each pump used 2 NEMA motors, one for the plunger and one for the valve). In our case, 7 pumps were used (14 drivers).

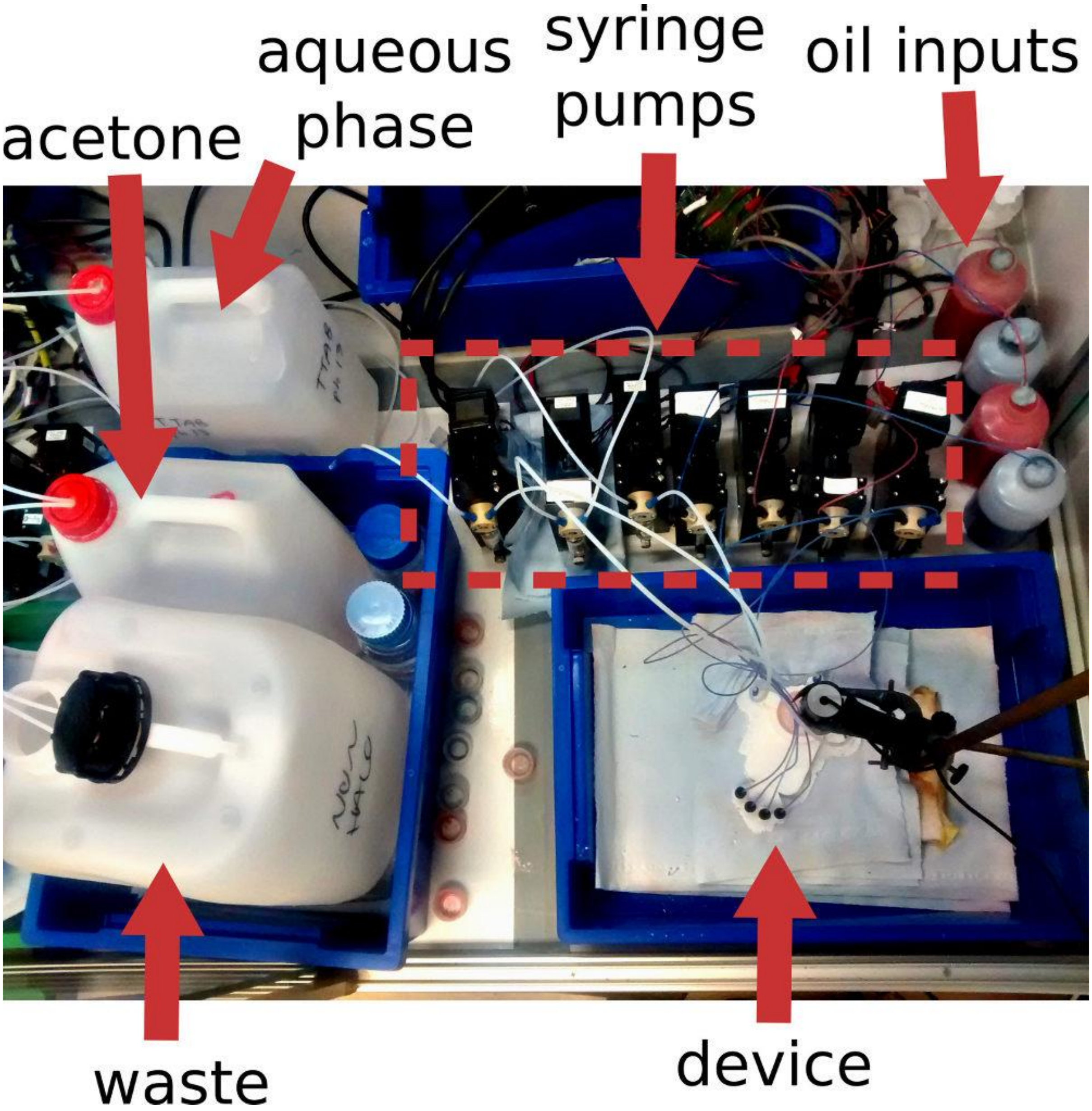

Supplementary Figure 6. Platform set-up. The device is connected to seven pumps, and each of these pumps is connected to one of the inputs: four oil inputs, aqueous phase, acetone, or waste output.

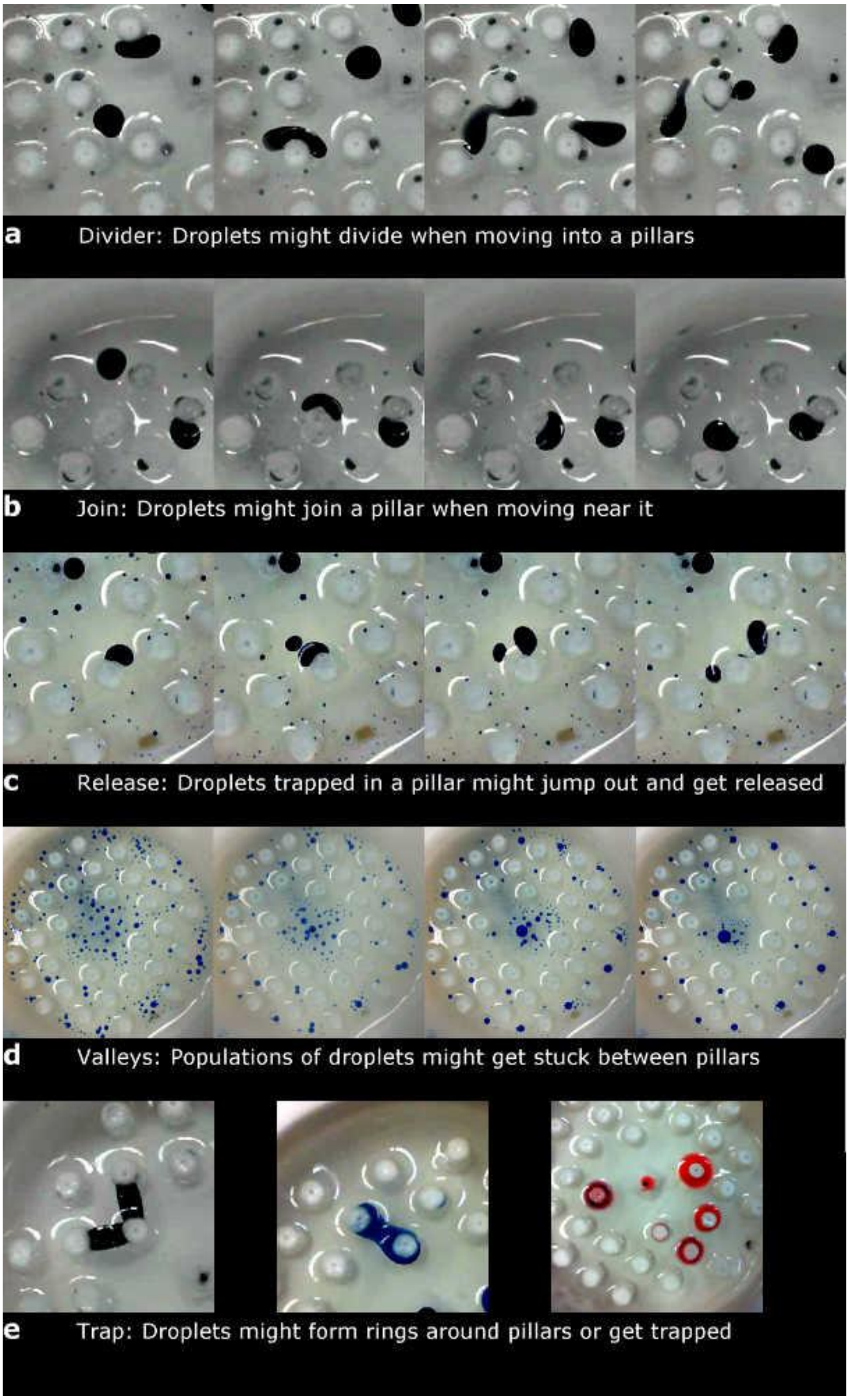

Supplementary Figure 7. Droplet interactions with the obstacles observed during the lattice search. Screenshots of the droplet behaviours as a function of time (left to right) except for (e) which represents single events.

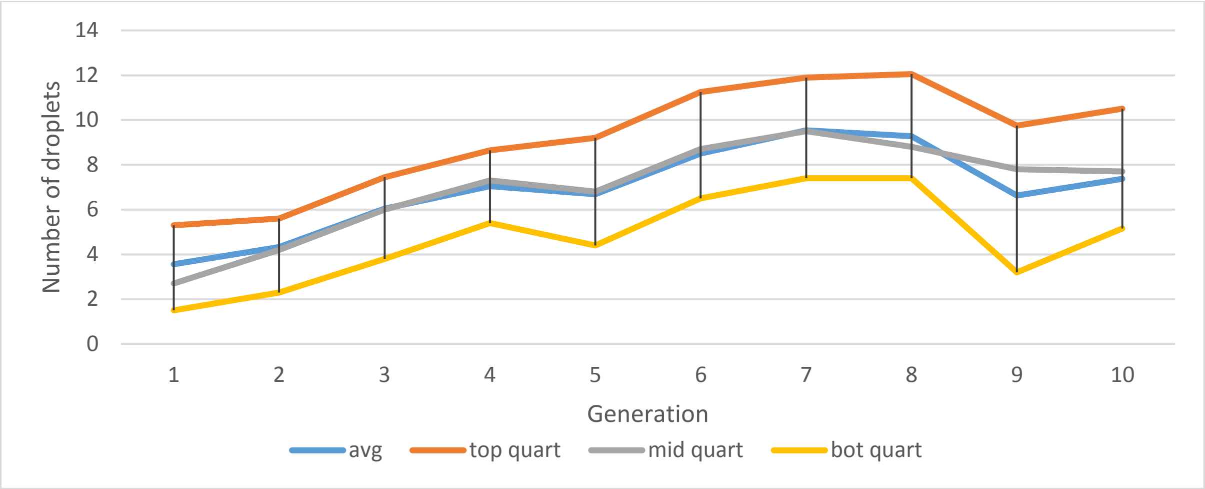

Supplementary Figure 8. Evolutionary trajectory of a Genetic Algorithm run using the empty environment. First GA run executed to validate the device, with the GA configuration as defined. There can be seen a drop in values between generation 8 and generation 9. This drop can be probably be attributed to the stochasticity of the algorithm, especially considering the low population size (20) and the high mutation rate used (10%).

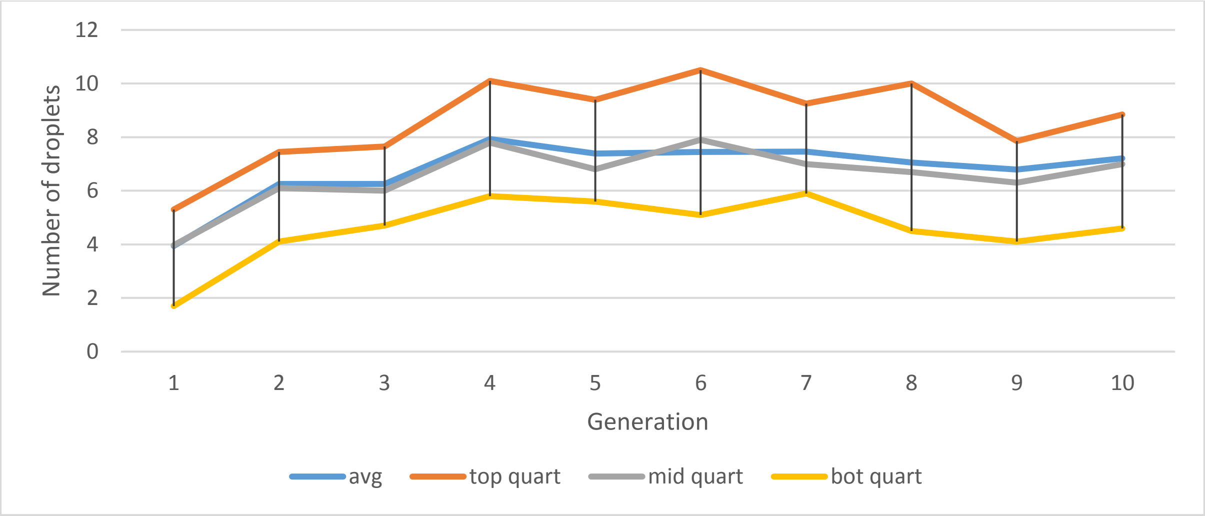

Supplementary Figure 9. Second evolutionary trajectory of a Genetic Algorithm run using the empty environment. Second GA run executed. The GA configuration is the same as before. In this case the growth flattened out after generation 4, although the final values almost doubled the initial values.

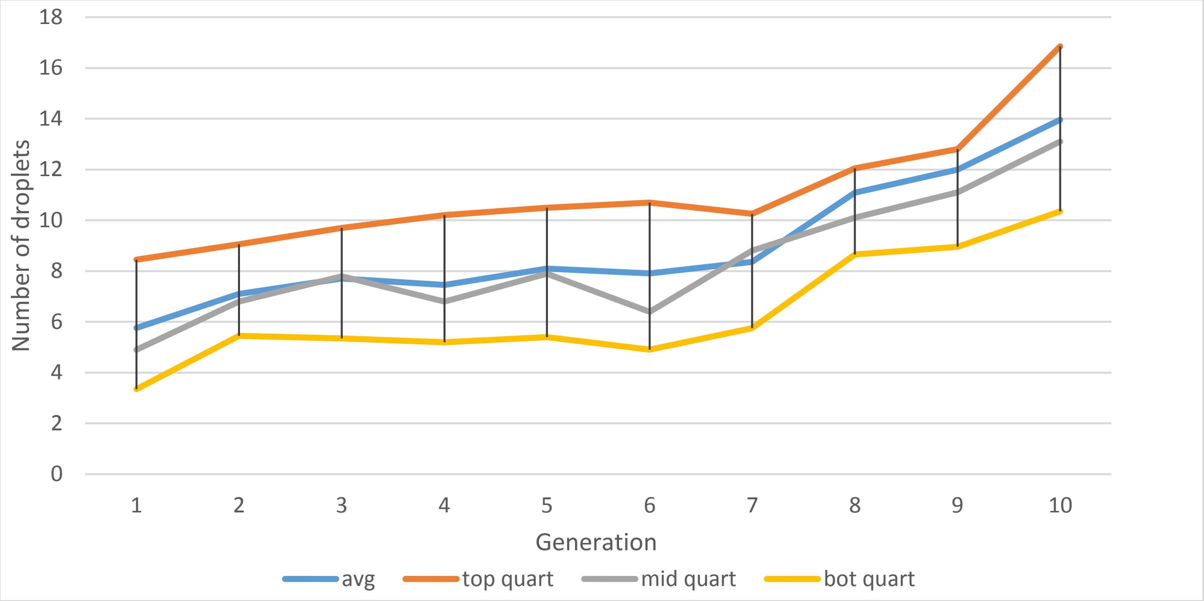

Supplementary Figure 10. Third evolutionary trajectory of a Genetic Algorithm run using the empty environment. Third GA run executed. The GA configuration is the same as before.

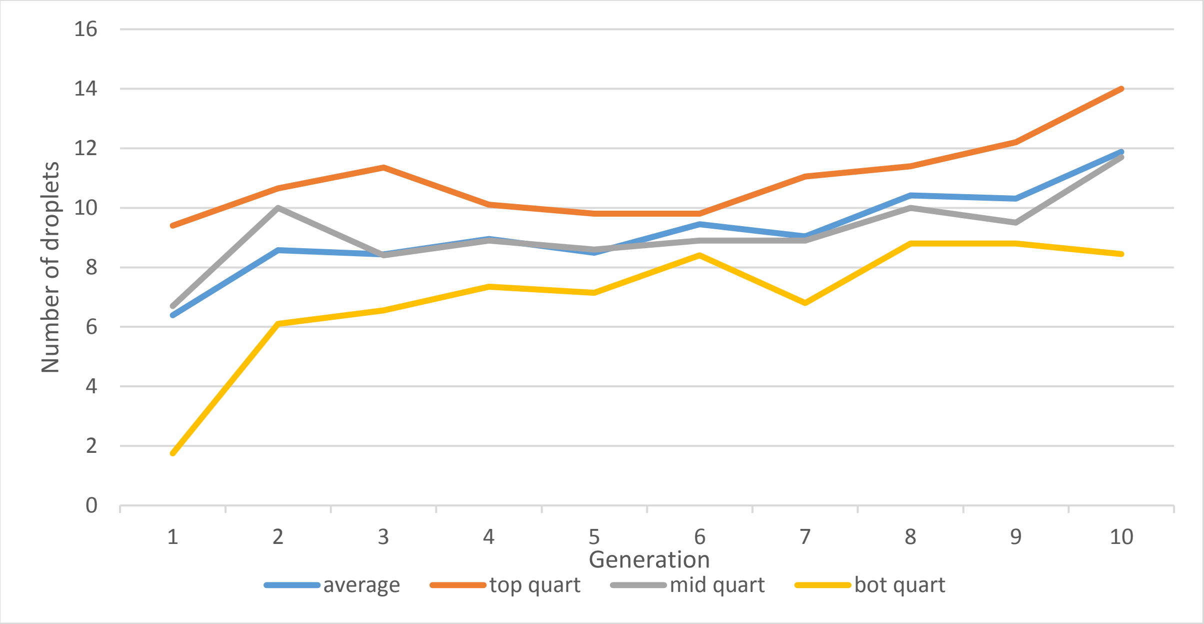

Supplementary Figure 11. Fourth evolutionary trajectory of a Genetic Algorithm run using the empty environment. Fourth GA run executed. The GA configuration is the same as before.

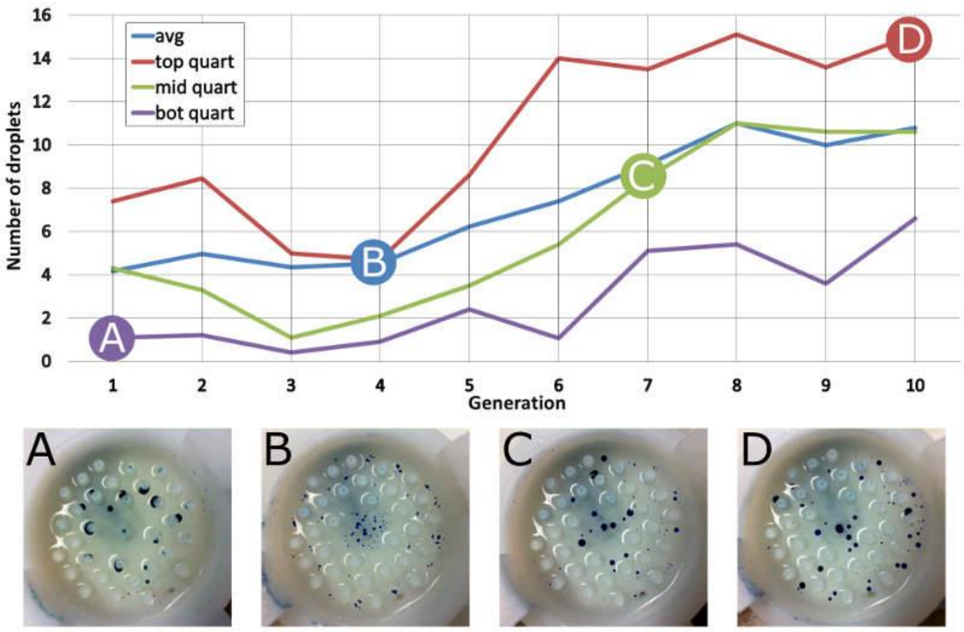

Supplementary Figure 12. Fitness increase over successive generations when using the pillars arena. Top: Linear plot showing the change in fitness (evolutionary trajectory) over each successive generation of experiments for the defined fitness function (droplet activity). Bottom: Using the pillars environment, successive pictures were taken to represent the increasing number of droplets through generations.

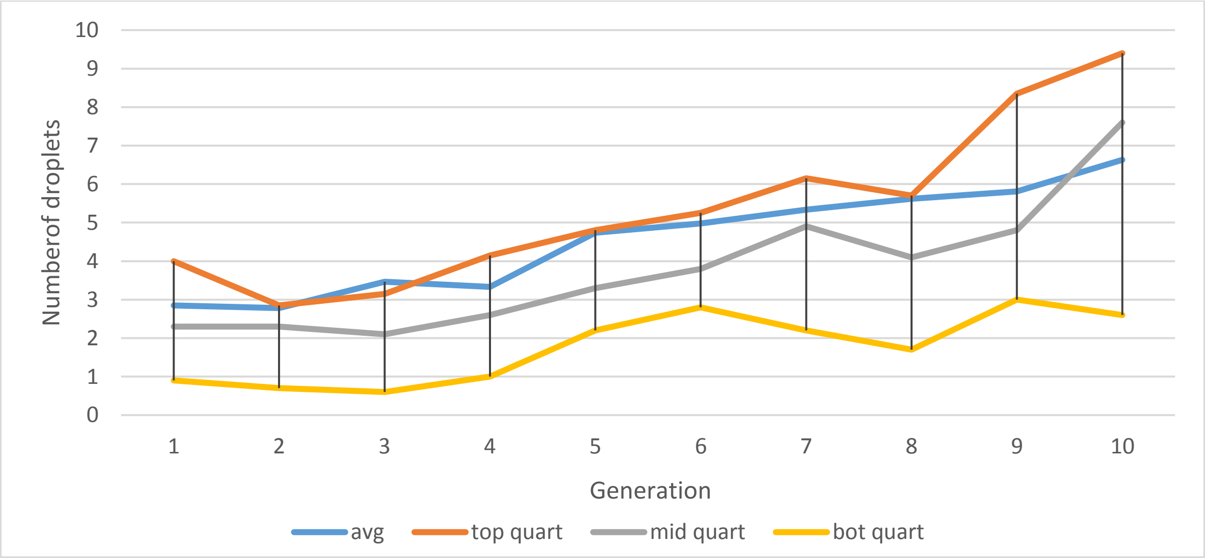

Supplementary Figure 13. Second evolutionary trajectory of a Genetic Algorithm run using the pillars environment.

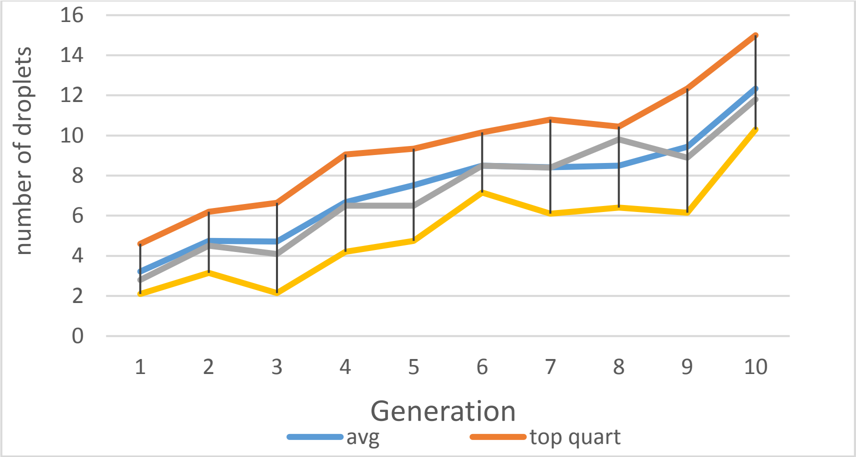

Supplementary Figure 14. Evolutionary trajectory of a Genetic Algorithm using the procedurally generated environment. First GA run performed to validate the environment. The fitness values grew steadily.

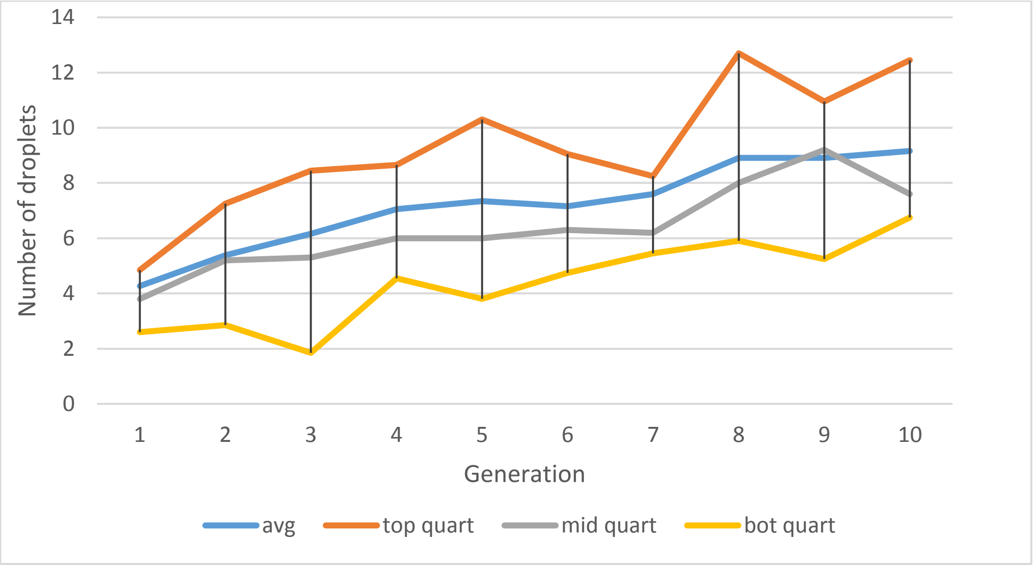

Supplementary Figure 15. Evolutionary trajectory of a Genetic Algorithm using the procedurally generated environment. Second GA run performed to validate the environment. The fitness values grew steadily.

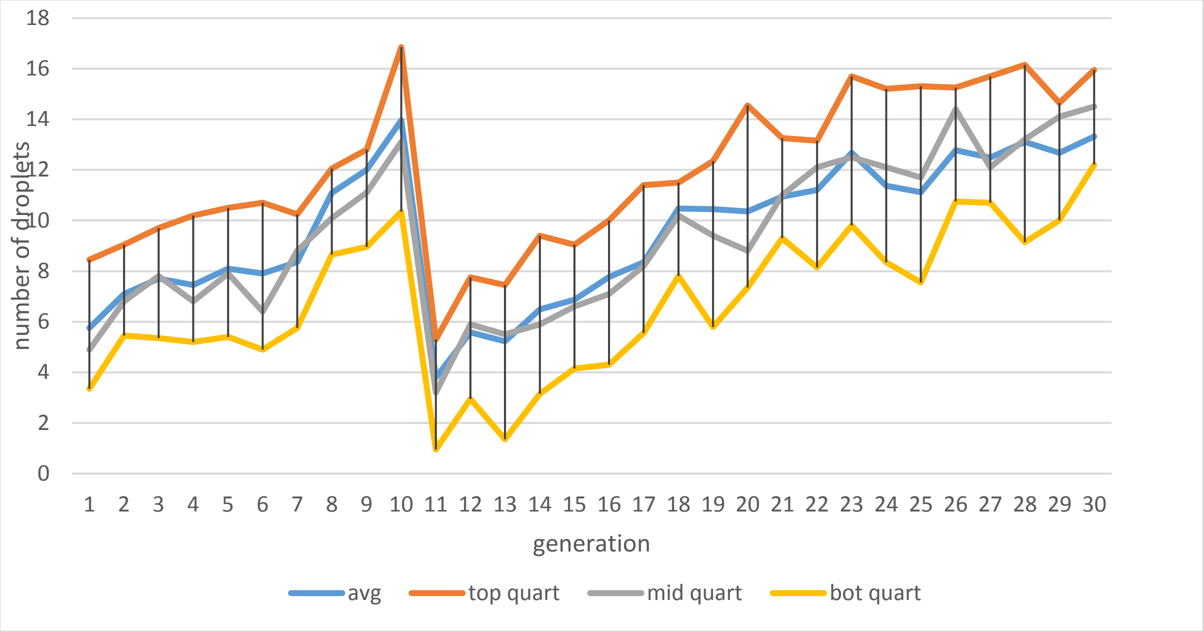

Supplementary Figure 16. Environmental change model. As in Figure 6 in the main manuscript, this linear plot represents the change in the evolutionary trajectories caused by changing the environment between generations. As before, the change was performed during generations and 10 and 11, and as it can be seen, the fitness value dropped by more than half. All the other GA conditions were exactly the same as in previous experiments.

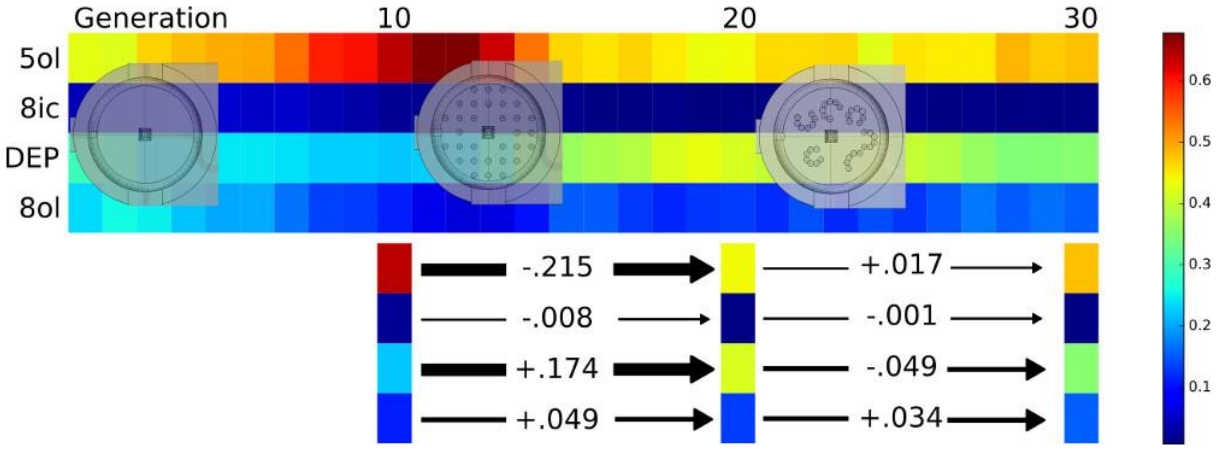

Supplementary Figure 17. Heatmap showing the genome evolution through 30 generations, where the first 10 used the empty environment, the following 10 used the environment populated with pillars, and the last 10 used the environment containing cave-like structures. Each of the columns in the heatmap represents a generation, and each of the rows represents one of the four components that formed our droplets. For each generation its average genome was calculated, and using this average value per component a color was assigned from the "jet" colormap. The bottom part of the figure focuses on the genomes at the last generation for each environment, where with bigger arrows significant changes are specified.

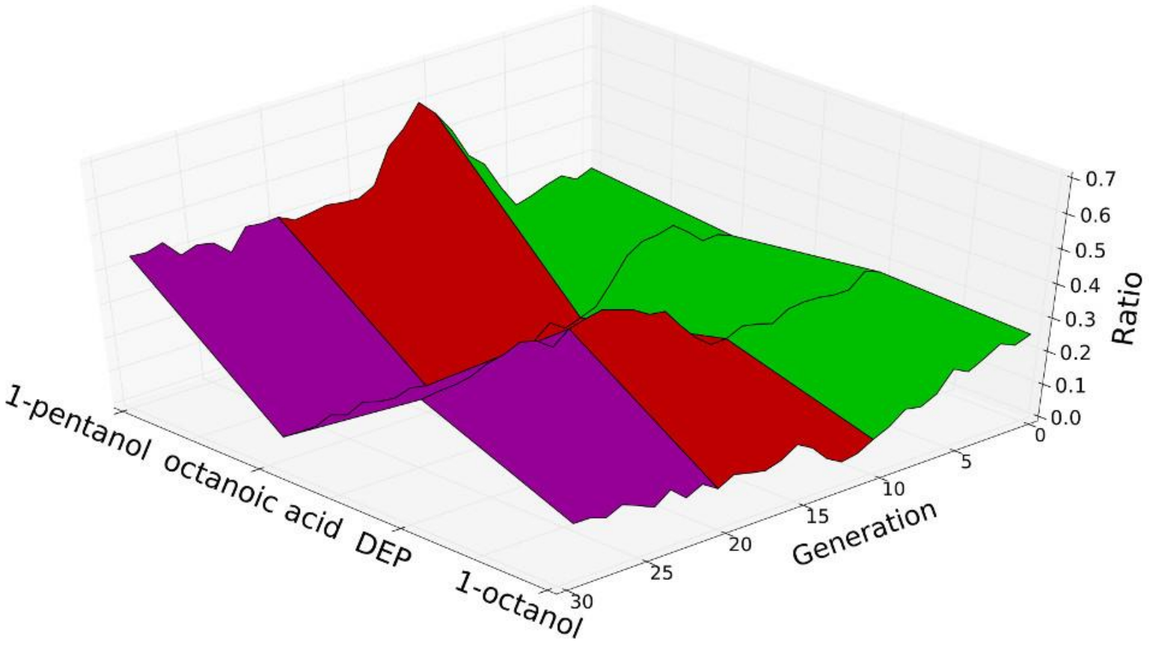

Supplementary Figure 18. Weighted average genome evolution for the hybrid GA run using the three environments in a consecutive way. The green area marks when the empty arena was used. The red area marks where the pillars arena was used. The purple area marks when the procedurally generated arena was used.

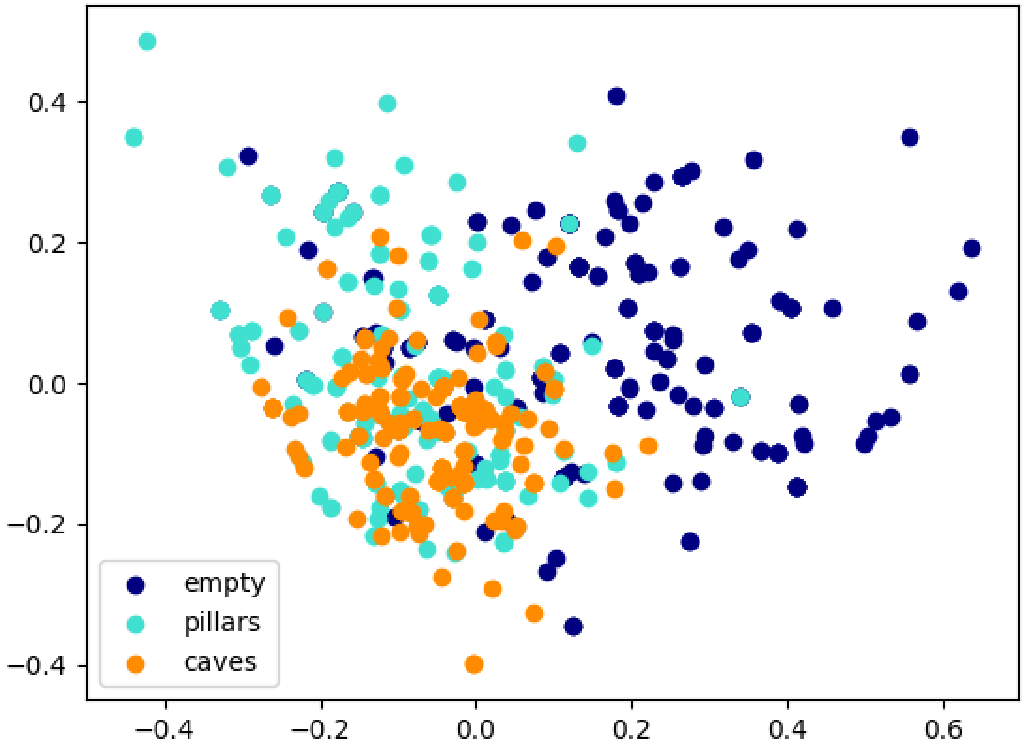

Supplementary Figure 19. PCA analysis of the data generated by the GA hybrid run with 30 generations. Each point represents a single recipe. The dimensionality of the original dataset was reduced to 2 and plotted here.

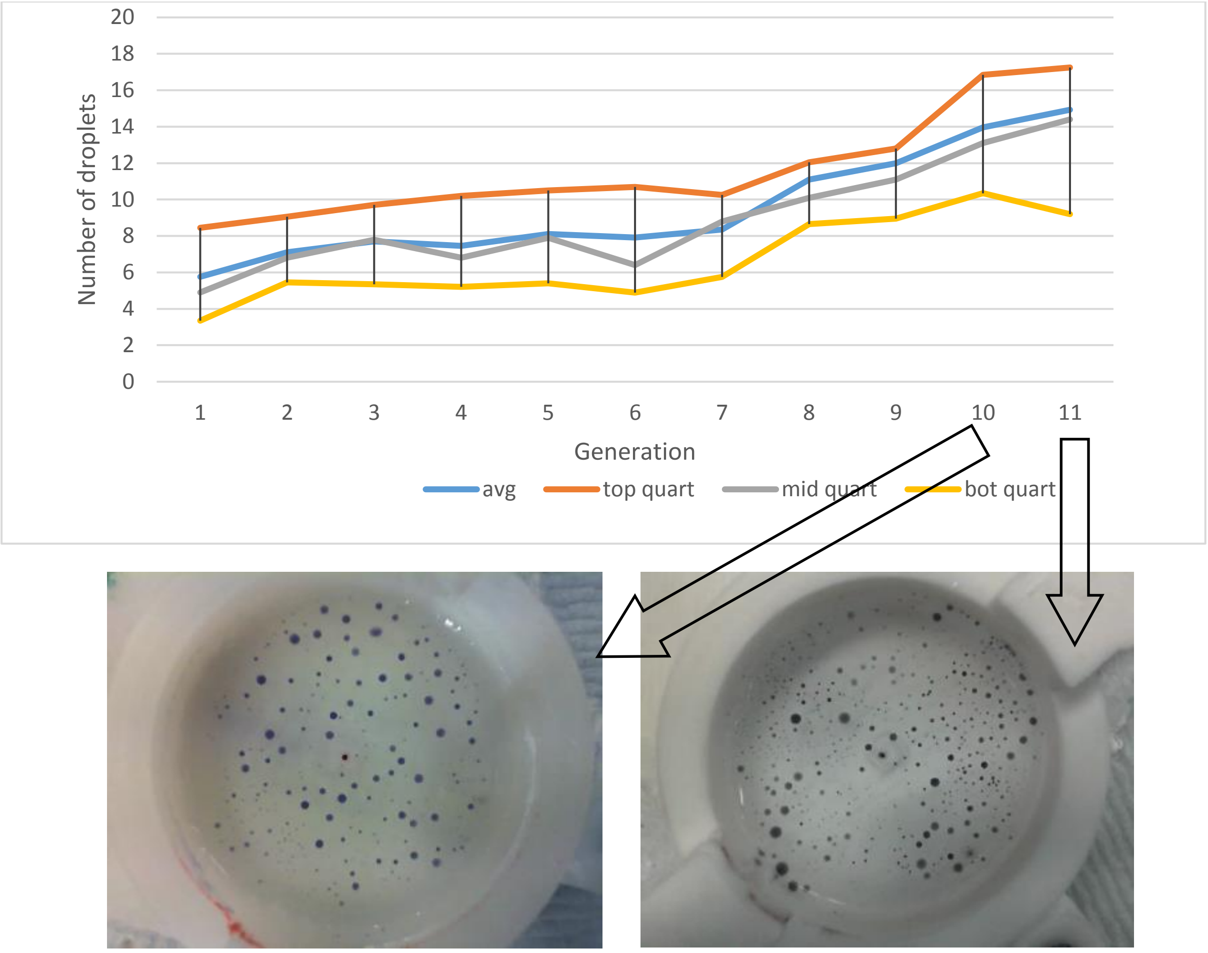

Supplementary Figure 20. Control test 1. After 10 generations using the empty environment as seen in the picture on the bottom left, the device was swapped by the device seen in the picture on the bottom right. The evolutionary trajectories hardly saw a difference. Also, the device used during the 10 first generations used “natural” PP, while the one in the right used “white” PP. This, this shows that the different types of PP did not make a difference.

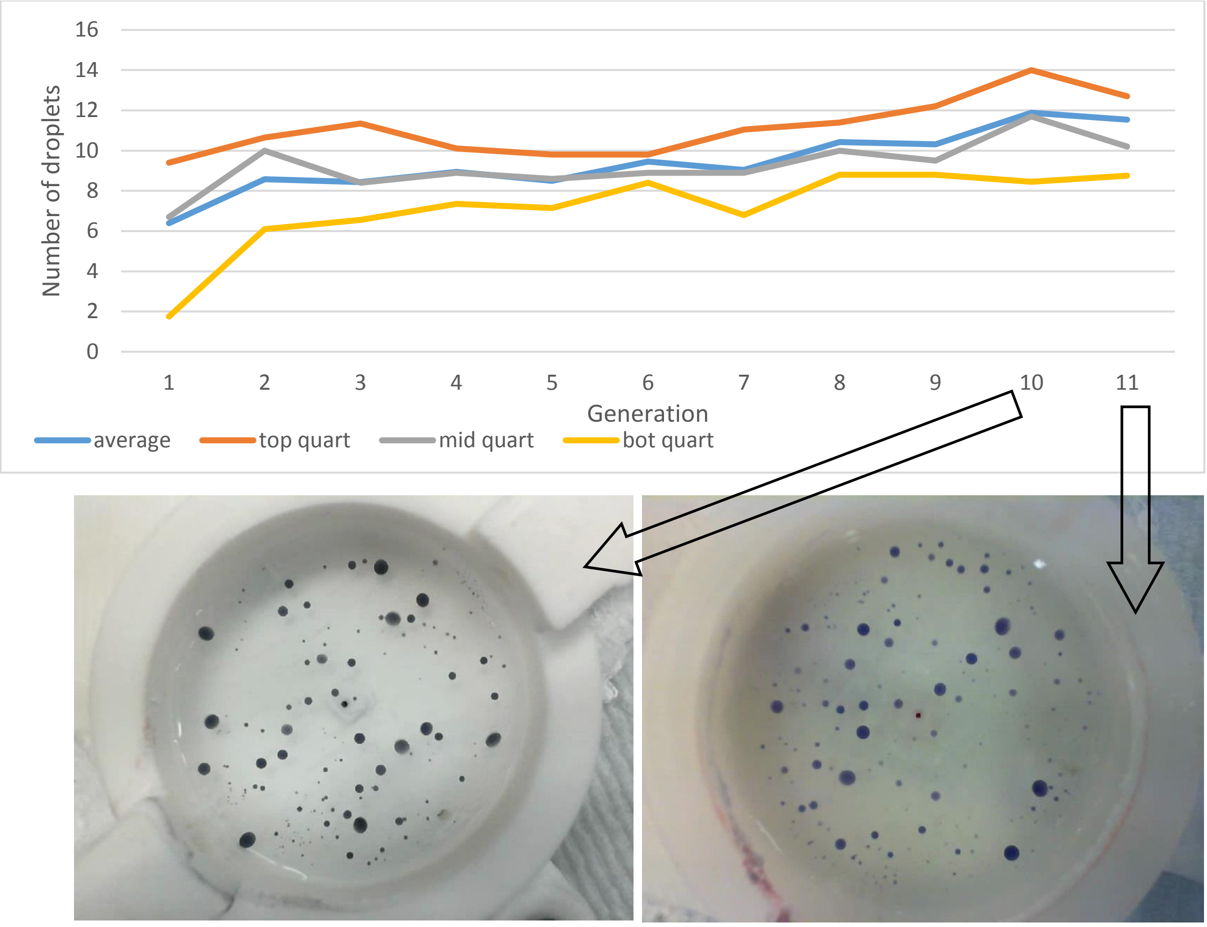

Supplementary Figure 21. Control test 2. Same as in Supplementary Figure 20. In this case, the first 10 generations used a device printed with “white” PP, and the eleventh generation used a device printed with “natural” PP. There were no major differences appreciated, which corroborates our hypothesis that the evolutionary changes are based on the different arenas used, and not in the individual characteristics of the used device.

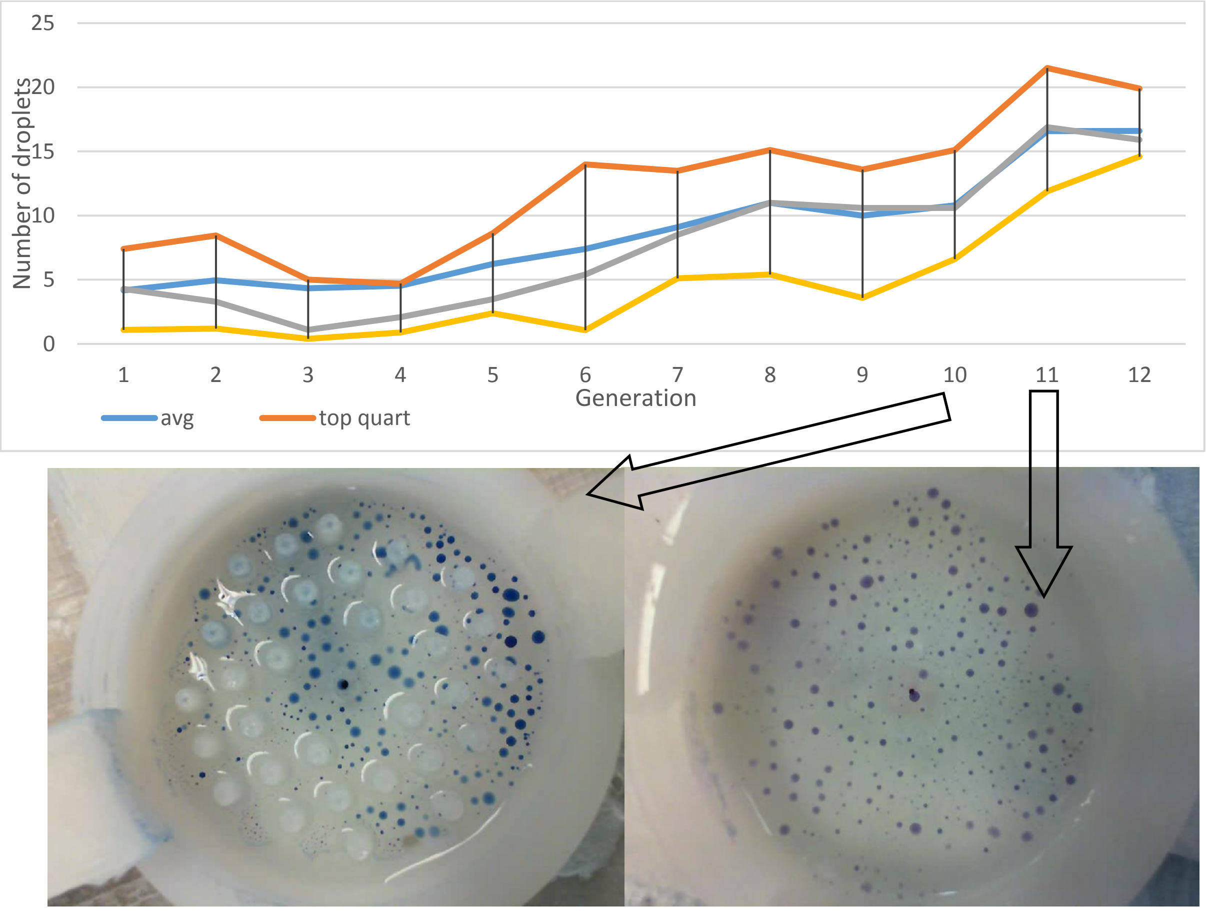

Supplementary Figure 22. Control test 3. Hybrid GA run in order to analyse the effect of removing the pillars from the environment. The removal was performed between generations 10 and 11. As it can be seen in the evolutionary trajectories, the fitness values grew slightly.

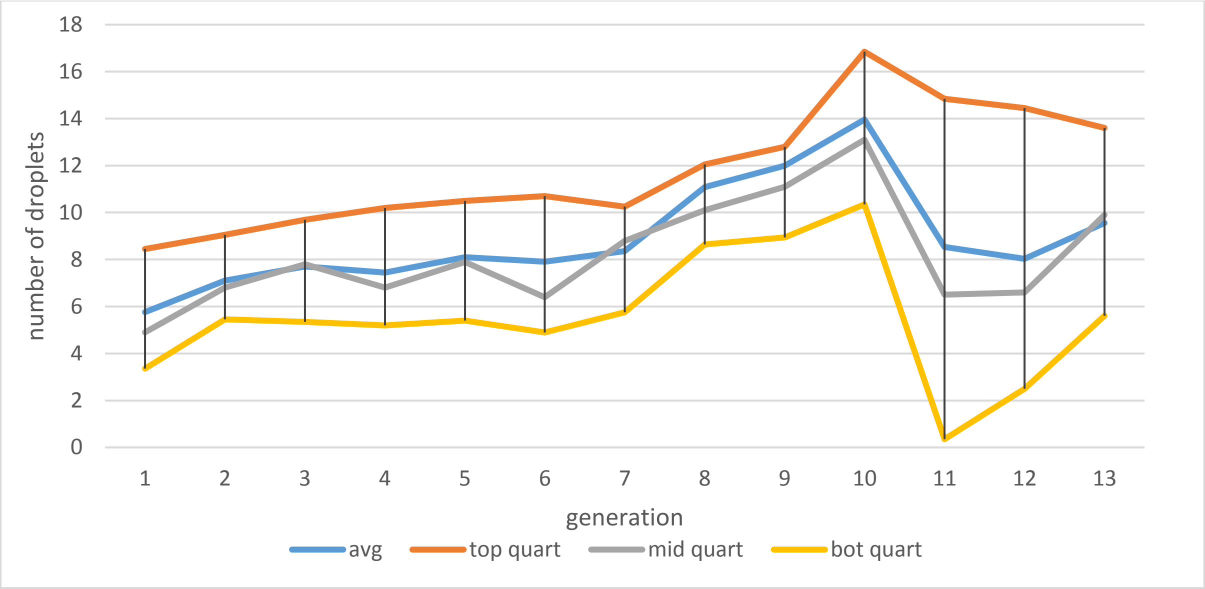

Supplementary Figure 23. Control test 4. Hybrid GA run where the first 10 generations used an empty arena, and from generation 11 onwards an arena with a procedurally generated environment was used. It can be seen that the evolutionary trajectories dropped, but not as much as they did when swapping an empty environment by one using a pillars arena. Also, the evolutionary trajectories recovered and grew again faster than before.

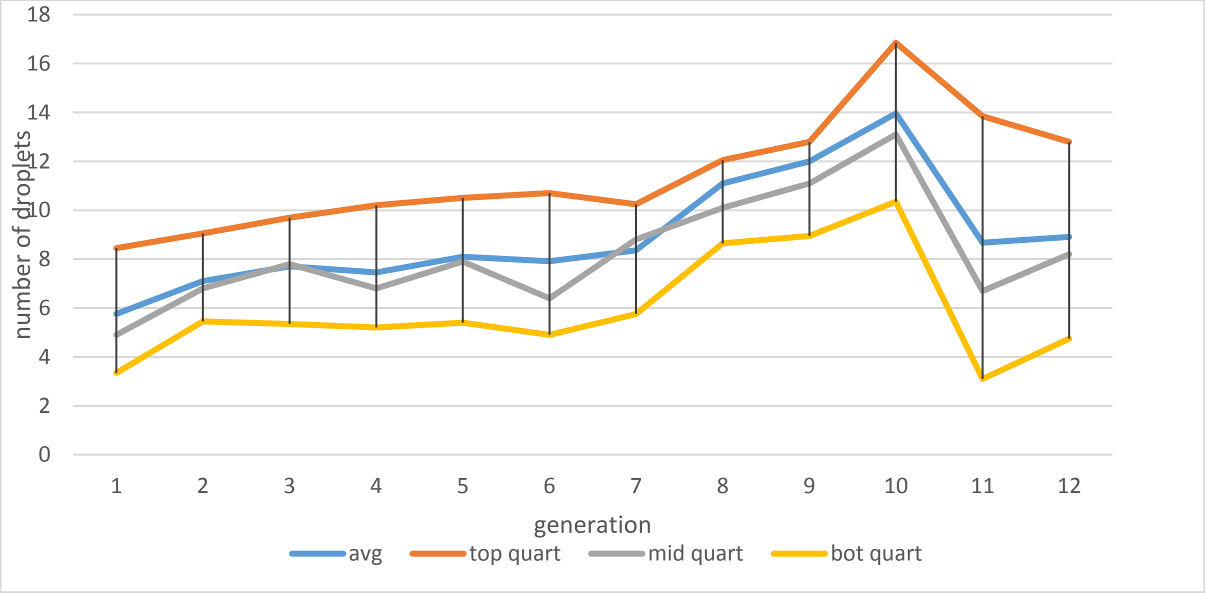

Supplementary Figure 24. Control test 5. Hybrid GA run where the first 10 generations used an empty arena, and from generation 11 onwards an arena with a procedurally generated environment was used. It can be seen that the evolutionary trajectories dropped, but not as much as they did when swapping an empty environment by one using a pillars arena. Also, the evolutionary trajectories recovered and grew again faster than before.

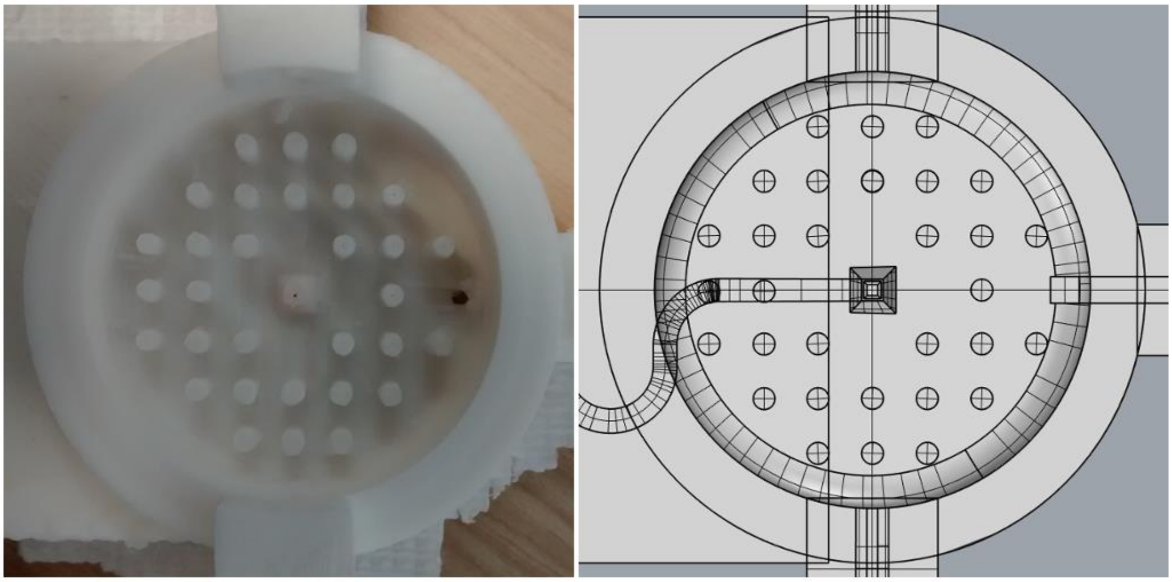

Supplementary Figure 25. Pillars arena. This arena was manually designed.

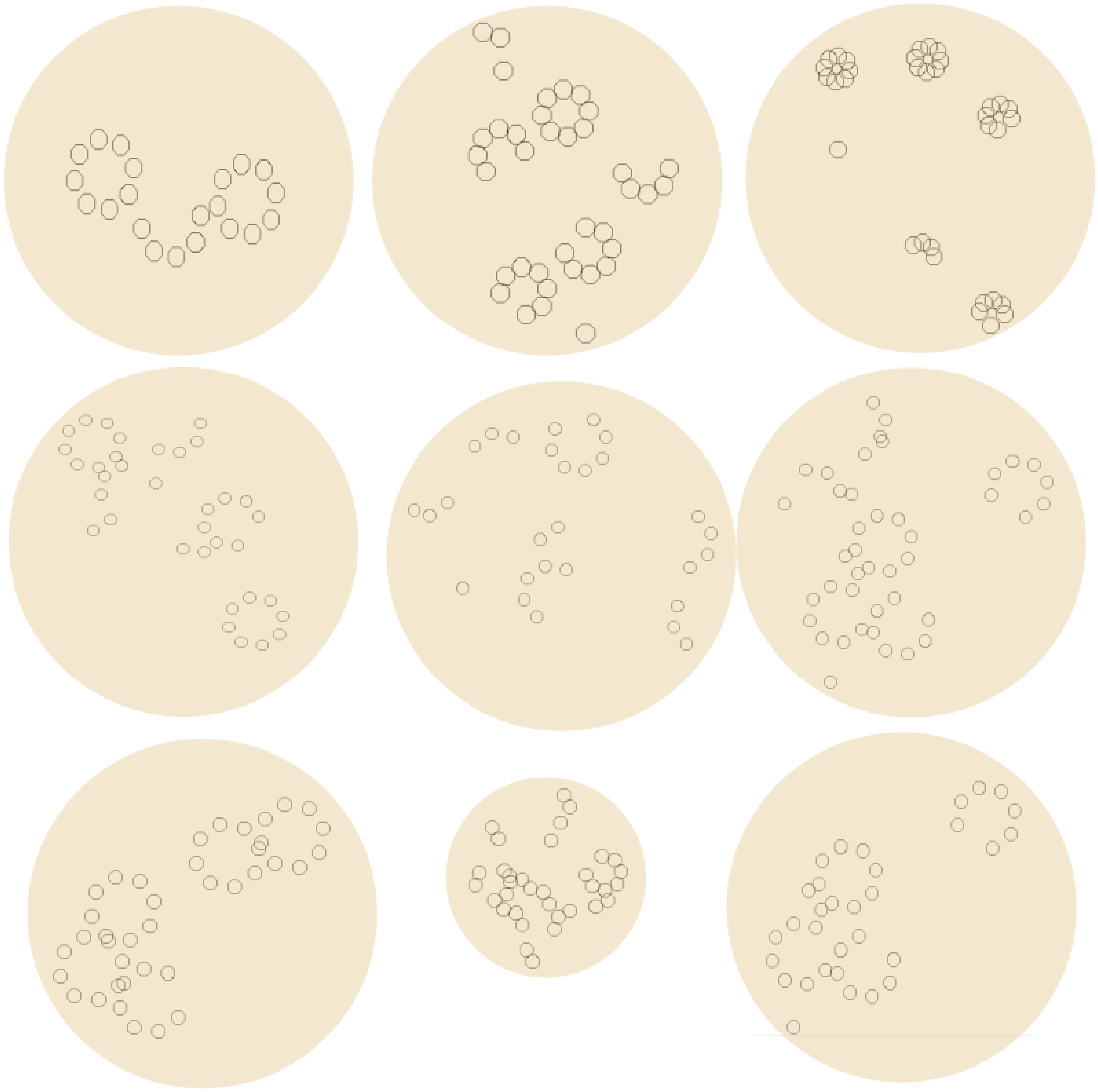

Supplementary Figure 26. Procedurally generated environments using a L-system.

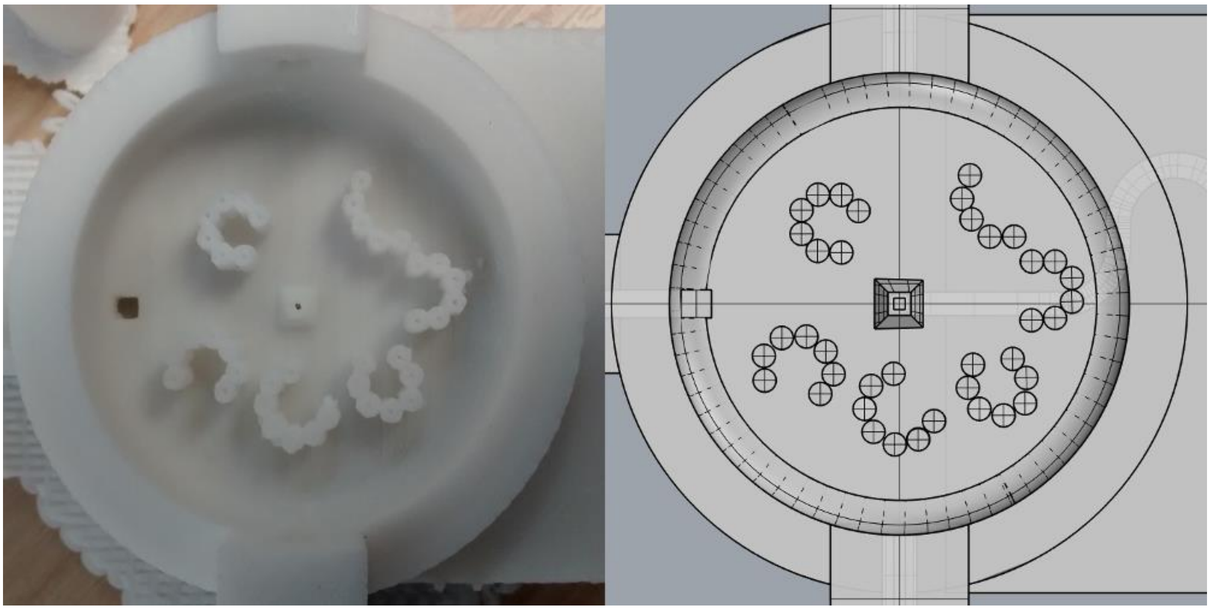

Supplementary Figure 27. Procedurally generated environment using a L-system. Left: The printed device with the actual environment. Only the pillars were part of the environment, everything else were artefacts from the 3D printing process. Right: 3D model of the design.

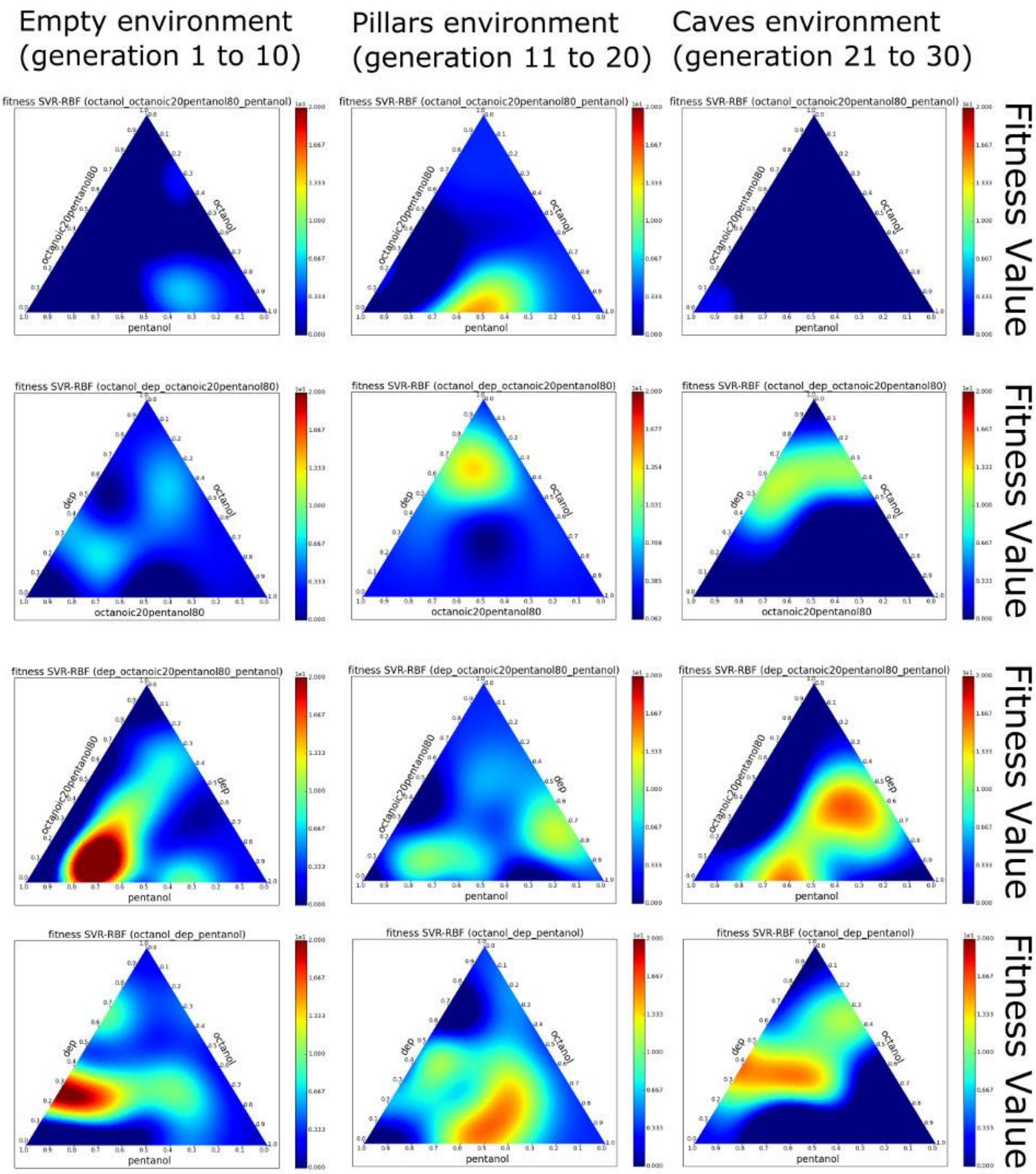

Supplementary Figure 28. Full fitness landscapes produced for the hybrid GA run with the SVR as described. The third row represents the ones used in Figure 4 of the main manuscript.

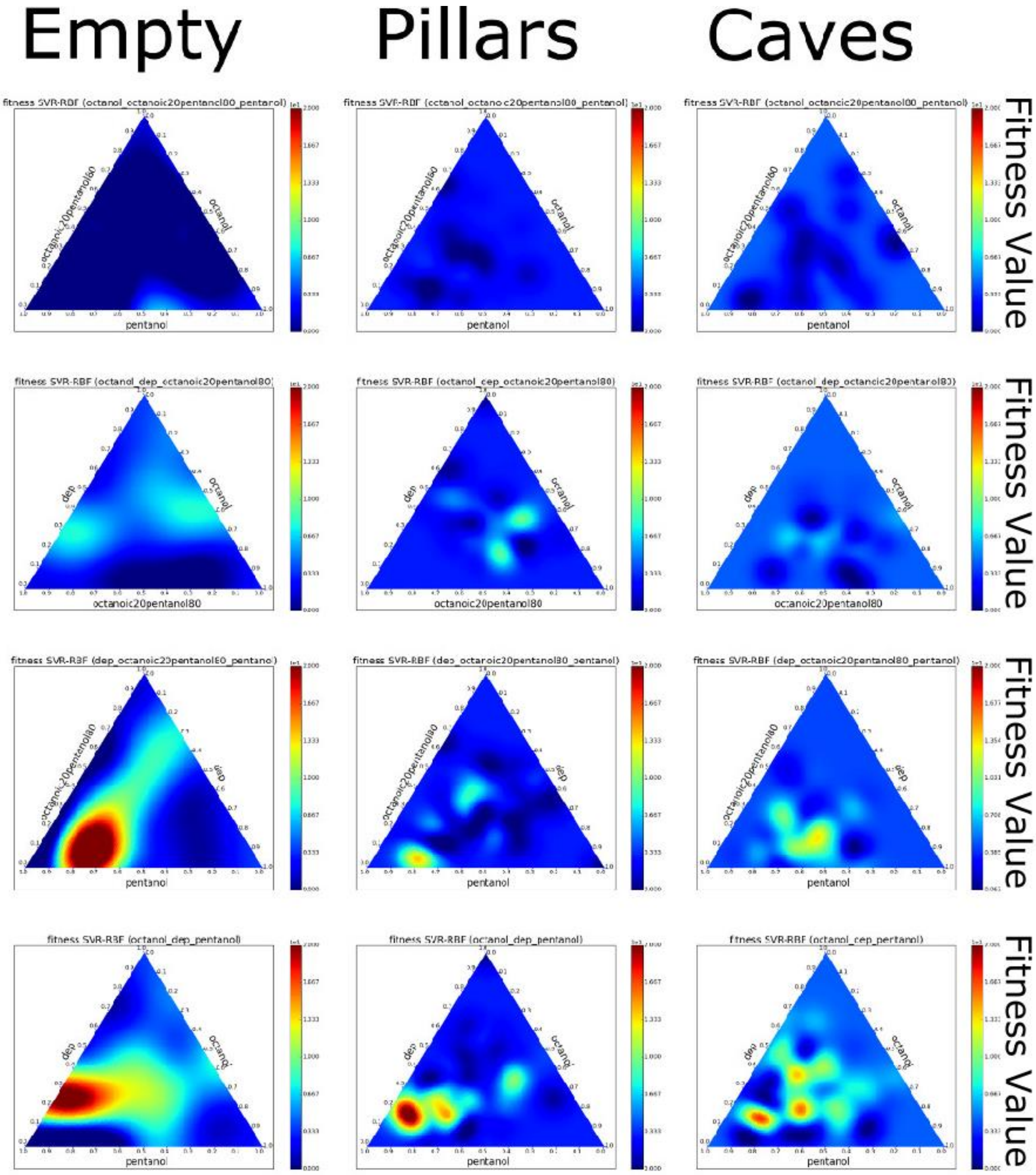

Supplementary Figure 29. Full fitness landscapes produced with the SVR as described.

Supplementary Table 1: Environments tested against best genomes

Droplets active at the end of experiment (median value):

	Tested in Empty	Tested in Pillars	Tested in Caves
Best from Empty	12.2	3.2	6.7
Best from Pillars	11.9	9.3	11
Best from Caves	14.4	6.8	13.4

Supplementary Table 2: Environments tested against best genomes

Following the previous table, and considering the diagonal as 0, and comparing the each row against its diagonal member:

	Tested in Empty	Tested in Pillars	Tested in Caves
Best from Empty	0	-9	-5.5
Best from Pillars	+2.6	0	+1.7
Best from Caves	+1	-6.6	0

Supplementary Note 1: Platform bill of materials

- Each device was designed using Rhinoceros CAD software, and 3D printed using the 3D printer "Bit from Bytes" using polypropene (PP) as thermopolymer.

- The syringe pumps used were "TriContinent C-Series". Three of them used 5 ml syringes, and four of them used 500 µl syringes. All of them used the same 3-way polyether ether ketone (PEEK) valves.

- The electronics from these pumps were replaced with custom-made PCBs in order to power them using an Arduino board.

- The stepper driver used to power the syringe pumps were Pololu a4988.

- An Arduino Due was used to control the syringe pumps.

- "IDEX Health Science PEEK 1/8" tubing was used to connect acetone, aqueous phase and waste from the containers to the syringe pumps, and from the pumps to the device.

- Flangeless fitting nuts, 1/8" OD Tubing, PEEK, were used to connect these tubes to the syringe pumps and device.

- “IDEX Health Science FEP Ora 1/16 x 0.20” was used to carry organic phases from reagent bottles to syringe pumps and from syringe pumps to the device.

- Flangeless fitting nuts, 1/16" OD Tubing, PEEK, were used to connect these tubes to the syringe pumps and device, with corresponding cone shaped fitting.

- A Microsoft LifeCam was used to record the experiments.
