# Supplementary material for: Adaptive artificial evolution of droplet protocells in a 3D-printed fluidic chemorobotic platform with configurable environments
Source: Nat Commun. 2017 Oct 26;8:1144. doi: 10.1038/s41467-017-01161-8 (PMC5658334; doi:10.1038/s41467-017-01161-8)
Supplement: Supplementary file 3 — Description of Additional Supplementary Information [file 41467_2017_1161_MOESM3_ESM.pdf]

Description of Additional Supplementary Files

File Name: Supplementary Movie 1
Description: Shows a trailer of the working platform.

File Name: Supplementary Movie 2
Description: Shows a video of how the droplet recipes evolve through a genetic algorithm.

File Name: Supplementary Movie 3
Description: Shows how the evolutionary fitness landscape evolves through a genetic algorithm.
